# 4095 Concurrent assessment of experimental pain and self-reported pain intensity with acute exercise intervention in fibromyalgia; clarifying or obscuring clinical outcomes?

**DOI:** 10.1017/cts.2020.294

**Published:** 2020-07-29

**Authors:** Giovanni Berardi, Grace Ptizen, Matthew DellaIacono, Marie Hoeger Bement

**Affiliations:** 1 Marquette University

## Abstract

OBJECTIVES/GOALS: Experimental pain testing is used to identify changes in nociceptive processing and outcomes with intervention. This study investigated exercise induced changes in experimental pain and self-reported pain intensity after an acute bout of exercise in participants with fibromyalgia. METHODS/STUDY POPULATION: Ten females with fibromyalgia (55±10yr) were familiarized to study procedures and underwent submaximal (20% maximal voluntary contraction) intermittent eccentric muscle contractions isolated to the right arm for 10-minutes. Self-reported pain intensity (0-10 numerical pain rating scale [NPRS]) of the exercising arm was measured before, during, and after exercise; whole-body pain intensity was measured before and after exercise. Experimental pain testing included measurement of pressure pain thresholds (kPa [PPTs]); temporal summation (TS) of pressure pain with a constant mechanical pressure; and TS of punctate pressure with repeated application of monofilaments before and after exercise. RESULTS/ANTICIPATED RESULTS: Participants reported minimal to moderate arm pain (3.1±2.1) during exercise. Following exercise, arm pain and whole-body pain significantly increased (3.1±2.2 and 1.6±0.5, respectively) [p<0.05]. No change occurred with PPTs at the bicep (138.9±49.5 to 142.8±55.3), PPTs at the quad (212.0±105.4 to 228.1±100.0), TS of mechanical pressure pain (7.6±2.1 to 7.9±1.5), TS of punctate pressure pain at the bicep (2.6±1.7 to 3.0±1.5), and TS of punctate pressure pain at the quad (3.6±1.5 to 3.7±1.4) before to after exercise respectively [p>0.05]. The change in self-reported arm and whole-body pain did not correlate with the change in experimental pain testing. DISCUSSION/SIGNIFICANCE OF IMPACT: In people with fibromyalgia, there is no relation between self-reported clinical pain and experimental pain following a single exercise session. Further research should identify the influence of exercise training on pain perception and if experimental pain testing translates to clinical insight.

